# Maltreatment in Childhood and Perceived Partner Responsiveness in Adult Romantic Relationships: A Dyadic Daily Diary and Longitudinal Study

**DOI:** 10.1177/10775595211057230

**Published:** 2021-12-05

**Authors:** Marie-Pier Vaillancourt-Morel, Natalie O. Rosen, Katherine Péloquin, Sophie Bergeron

**Affiliations:** 1 14847 Université du Québec à Trois-Rivières, Trois-Rivières, QC, Canada; 2 3688 Dalhousie University, Halifax, NS, Canada; 3 IWK Health Centre, Halifax, NS, Canada; 4 5622 Université de Montréal, Montréal, QC, Canada

**Keywords:** childhood maltreatment, romantic relationships, perceived partner responsiveness, dyadic daily diary, longitudinal analysis

## Abstract

This study examined the associations between childhood maltreatment (CM) and the mean-level of perceived partner responsiveness (PPR; the extent to which individuals feel cared for, understood, and validated by their partner) over 35 days, the day-to-day variability in PPR, and the initial levels and trajectories of PPR over 1 year in community couples. Both members of 228 couples completed a self-reported measure of CM and provided daily reports of PPR over 35 days and retrospective reports of PPR at three time points over 1 year. A person’s greater CM was related to a lower mean level of PPR over 35 days and to a lower initial level of their own PPR. A person’s sexual abuse, physical neglect, and emotional neglect had an effect over and above other forms of CM in these associations. A person’s greater CM was also related to higher day-to-day variability in their own and their partner’s PPR, and a person’s greater emotional neglect was associated with a sharper decrease over time in their own PPR. These findings provide a more fine-grained understanding of how CM may affect the perceptions of being cared about, accepted, and validated by a partner on a daily basis and over time.

Perceived partner responsiveness (PPR) has been identified as a central process to understand close relationships (Reis, 2012). PPR is conceptually distinct from partner support, which has more to do with the quantity of different types of support received, whereas PPR refers to the degree to which individuals believe that their romantic partner cares about, understands, and validates their thoughts and feelings (Reis et al., 2004). PPR is associated with better mental and physical health including higher psychological well-being and lower all-cause mortality (Selcuk & Ong, 2013; Tasfiliz et al., 2018). Greater PPR is also related to a host of romantic relationship benefits such as greater relationship satisfaction and intimacy, as well as sexual function, satisfaction, and desire in different clinical and general population samples (Bergeron et al., 2021; Birnbaum et al., 2016; Laurenceau et al., 2005).

Perceptions of others are often biased (Kenny & Acitelli, 2001), and indeed, PPR and observed partner behaviors are only weakly correlated (*r* = .01–.30; Birnbaum et al., 2016). Although partner empathic responses can be objectively coded as responsive or unresponsive, what matters most is how a person interprets the response, as it is the perception that is related to a host of romantic relationship and health benefits (Reis et al., 2004). A key pathway to promote interactions in which partners feel understood, validated, and cared for involves identifying factors that distort the perception of partner responsiveness (Reis, 2017). The responsiveness model suggests that the perceiver’s interpretive filter derive from experiences in the present and in other recent or distal relationships (Reis, 2017).

One such distal interpersonal experience that may affect PPR is child maltreatment (CM), which includes physical, emotional, and sexual abuse as well as physical and emotional neglect. Theory and research evidence the negative effects of CM on self and other representations (Bowlby, 1969; Briere, 2002) and it is known to be related to a host of difficulties in adult romantic relationships (Colman & Widom, 2004; Godbout et al., 2019). Although one study found a significant cross-sectional association between CM and PPR (Vaillancourt-Morel et al., 2019), we know little about the unique associations with each form of CM and how CM is related to PPR from day-to-day and over the course of a relationship. Such knowledge would allow a more fine-grained understanding of how CM may affect PPR in daily life and over time, to guide the development of interventions that facilitate the experience of PPR. This study examined the associations between five forms of CM and mean-level of PPR over 35 days, day-to-day variability in PPR, and trajectories of PPR over 1 year in community couples.

## Childhood Maltreatment

CM refers to all forms of abuse and neglect that children under 18 years of age may experience at the hands of a caregiver (World Health Organization, 2016). In large population-based studies, 35–40% of individuals retrospectively report at least one form of CM, with multiple chronic victimizations being the norm (Cyr et al., 2013). CM is a relational trauma, whereby the betrayal, powerlessness, or disregard experienced have the potential to disturb future romantic relationships (Briere, 2002; Colman & Widom, 2004). Although studies suggest that some individuals with a CM history retain relatively stable and healthy romantic relationships (Domhardt et al., 2015), emerging evidence suggests that all forms of CM are associated with difficulties in several aspects of romantic relationships. Specifically, CM is related to intimate partner violence (Godbout et al., 2019), lower trust (DiLillo et al., 2009), sexual difficulties and dissatisfaction, and ultimately lower relationship satisfaction and relationship dissolution (Colman & Widom, 2004; Vaillancourt-Morel et al., 2015, 2021).

## Childhood Maltreatment and Perceived Partner Responsiveness

Given the central role of PPR in couple functioning and the numerous negative effects of CM on romantic relationships, it is surprising that we know so little on the CM-PPR association. The hypothesis that CM may affect perceptions of partner responsiveness is consistent with trauma theories, such as the self-trauma model (Briere & Scott, 2014). From this perspective, growing up in an impoverished social and emotional environment may strongly distort individuals’ internal representations of the self, others, and relationships, leading victims to view the self as shameful, helpless, and not deserving of love, and to perceive others, particularly a significant other, as inherently intrusive, rejecting, or abusive (Bowlby, 1969; Briere, 2002). Thus, romantic relationships may activate CM-related negative attributional styles, which might lead to biases such as greater levels of distrust in partners, fears of losing the partner, and potentially, lower or unstable perceptions of partner responsiveness (Briere, 2002; Gibb, 2002).

Past studies have shown that CM is related to the ways a person perceives the events in their lives. In a systematic review, emotional and sexual maltreatment were related in adulthood to the tendency to infer negative characteristics to events (Gibb, 2002). The romantic relationship might be the interpersonal context that could most strongly elicit CM-related perceptions, as CM occurs in caregiving relationships where trust, vulnerability, and betrayal may mimic those experienced in intimate relationships. Moreover, described as the secondary trauma effect, research has shown that the difficulties reported by CM victims are also reported by their partners, including lower relationship and sexual satisfaction (Nelson & Wampler, 2000; Vaillancourt-Morel et al., 2021; Whisman, 2014). In a sample of 156 couples, a cross-sectional study showed that perceptions of partner negative affective responses were biased by both participants’ own and their partner’s emotional abuse (Maneta et al., 2014). This study supports the strength of using dyadic data to examine whether CM may affect their partner.

In the only study examining the association between CM and PPR, women’s and men’s cumulative CM were related to their own lower levels of PPR among a sample of 346 mixed-sex couples (Vaillancourt-Morel et al., 2019). However, this study assessed PPR at only one time point. This is an important limitation given PPR has been shown to change from day-to-day and exhibit a small decline on average over the course of romantic relationships (Derrick et al., 2013; Gunaydin et al., 2020; Stanton et al., 2019). Moreover, change in PPR between time points was an important predictor of health indicators over time (Derrick et al., 2013; Stanton et al., 2019), and average PPR (mean PPR during the 3-week period) as well as PPR variability (consistency of PPR during the 3-week period) each uniquely predicted different components of romantic attachment (Gunaydin et al., 2020). Thus, we need to understand not only what is related to PPR at a given time point, but also how it is related to changes across time and day-to-day.

CM has been shown to have a small significant effect on patterns of change of marital and sexual satisfaction in romantic relationships (DiLillo et al., 2009; Vaillancourt-Morel et al., 2021). On average, as the relationship progresses, commitment increases, conflicts emerge, and partners’ show more vulnerability, which may trigger CM-related negative attributional styles that were not apparent before, and may disturb how victims perceive their partner over time (MacIntosh, 2017; Vaillancourt-Morel et al., 2016). The scarce daily diary studies focusing on CM have examined the effects on daily worries, positive thoughts, and emotional reactivity and indicated that CM is related to greater day-to-day variability in negative and positive affects (Arbel et al., 2018; Infurna et al., 2015). Although no study examined the associations with daily fluctuations in the perceptions of others, CM is related to a tendency to evaluate others with more polarity, alternating between idealization and devaluation (Showers et al., 2006; Twomey et al., 2000).

The only study that examined the CM-PPR association included cumulative CM exclusively (Vaillancourt-Morel et al., 2019), which gives limited information concerning the unique association between each form of CM and PPR. In fact, most research to date examining the effects of CM on romantic relationships examined either only a single form (Maneta et al., 2014; Vitek & Yeater, 2020), the cumulative score (Vaillancourt-Morel et al., 2019), or all forms of CM in separate models without controlling for the overlap among the forms (DiLillo et al., 2009; Vaillancourt-Morel et al., 2021). These designs do not allow conclusions about which form of CM has the strongest *unique* contribution. Consistent with complex trauma theories (Briere, 2002; Courtois, 2004), CM chronicity and co-occurrence have been consistently related to poorer relationship outcomes (Labella et al., 2018; Vaillancourt-Morel et al., 2019). However, the few studies examining all forms of CM concurrently yielded mixed findings; some suggested that abuses would be associated with worse outcomes (Labella et al., 2018; Lacelle et al., 2012) and others showed that emotional CM (i.e., emotional abuse and neglect) has specific and independent consequences given its pervasiveness (Gibb et al., 2001; Hildyard & Wolfe, 2002). Thus, it remains unknown whether one form of CM is uniquely associated with romantic relationship functioning including PPR.

### Current Study

The present daily diary and longitudinal study examined the associations between CM and the mean-level of PPR over 35 days, day-to-day variability in PPR, and the initial levels and trajectories of PPR over three time points across 1 year in community couples. We examined both cumulative CM and the unique associations between each of the five forms of CM and PPR. We hypothesized that cumulative CM would be related to lower mean-level, higher day-to-day variability, lower initial level, and sharper decrease over time in participants’ own PPR. Given mixed findings reported in past studies, we examined all five forms concurrently and partner effects in an exploratory manner. As this study included a wide range of relationship durations, we added relationship duration as a covariate.

## Method

### Participants

Participants were recruited through online advertisements, email lists, and flyers distributed in public places in two metropolitan Canadian cities. Advertisements informed participants about an online study on how sex and relationship intimacy contribute to the well-being of couples. Interested participants were contacted by a research assistant for a brief telephone eligibility interview. Both partners had to be at least 18 years of age, living together for at least 12 months, and sexually active at least once a month over the past 3 months. Couples were not eligible if one partner was pregnant or breastfeeding, was unable to comprehend either French or English, reported a severe mental or physical illness that affected their sexuality, or took prescribed medications regularly that affected their sexuality.

Of the 519 couples who contacted the research team, 254 (48.9%) could not be reached, were not eligible, or had one or both partners who were not interested in participating, 30 (5.8%) dropped out during the Time 1 survey, five (1.0%) failed two out of three attention questions in the Time 1 survey, and one (.2%) asked that their data be removed from the study. Thus, 229 couples were invited for the daily diaries and the longitudinal follow-ups. For the daily diaries, 11 (2.1%) additional couples dropped out before starting the daily diaries or during the first 2 days, and one (.2%) was excluded because of an error in data collection; resulting in a final sample of 217 couples (434 participants) for the daily diaries. For the longitudinal follow-up, 19 (3.7%) couples had separated at the Time 3 assessment. Data from these 19 couples were excluded from the longitudinal analyses as they could not be handled using the missing-at-random assumption because the separation could be associated with the couple’s PPR over time; this resulted in a sample size of 210 couples (420 participants) for the longitudinal follow-up. Of the 229 couples invited for the daily diaries and the follow-ups, only one couple was excluded in both the daily diaries and the longitudinal follow-up. Thus, sample characteristics are reported for the 228 couples (*n* = 456 participants) that were included in at least one part of this study.

This sample (*n* = 456) included 239 cis or trans women (52.4%), 189 cis or trans men (41.4%), and 28 nonbinary, queer, or gender fluid individuals (6.1%). Participants ranged in age from 18 to 70 years (*M* = 30.44, *SD* = 8.43). The majority of participants described their cultural identity as French Canadian (38.4%; *n* = 175) or English Canadian (37.1%; *n* = 169), followed by American (11.0%; *n* = 50), European (4.8%; *n* = 22), and a range of other cultural identities (9.6%; *n* = 44; First Nations, African, Asian, Middle Eastern, Latin American, Caribbean, mixed cultural identities, and none). On average, participants reported 16.61 years of education (*SD* = 2.92) which corresponds to a college undergraduate degree. Most participants reported an average annual personal income of less than $40,000 CAD (61.0%; *n* = 278); 27.4% reported between $40,000 and $69,999 (*n* = 125); and 11.6% reported more than $70,000 (*n* = 53). About half of participants defined their sexual orientation as heterosexual (55.0%; *n* = 251), with 10.7% (*n* = 49) identifying as bisexual, 18.6% (*n* = 85) as gay/lesbian, 8.8% (*n* = 40) as queer, 4.2% (*n* = 19) as pansexual, .9% (*n* = 4) as uncertain or confused, .2% (*n* = 1) as asexual, and 1.5% (*n* = 7) as “something else.” Couples had been in their current relationship from 1 to 37.83 years (*M* = 5.84, *SD* = 5.10). Most couples were living together without being married (72.4%; *n* = 165) and 27.6% were married (*n* = 63). Using the recommended cut-off scores to dichotomize the Childhood Trauma Questionnaire (CTQ) continuous scores on each subscale, among the 456 participants, 65.1% (*n* = 297) reported at least one type of CM: 18.0% (*n* = 82) reported physical abuse, 38.2% (*n* = 174) emotional abuse, 28.1% (*n* = 128) physical neglect, 49.1% (*n =* 224) emotional neglect, and 17.5% (*n* = 80) sexual abuse. The couples excluded only for the daily analysis reported a lower number of years of education (*n* = 22, *M* = 14.68 years, *SD =* 3.87) compared with those that were included (*n* = 434; *M* = 16.71 years, *SD =* 2.84), *t* (454) = −3.21, *p* < .001. The couples excluded only for the longitudinal analysis were significantly younger (*n* = 36, *M* = 26.64, *SD =* 6.84) than intact couples (*n* = 420, *M* = 30.77, *SD =* 8.48), *t* (454) = 2.84, *p* = .005. There were no other significant differences on sociodemographic variables.

### Procedure

All procedures were approved by Université de Montréal and Dalhousie University’s Institutional Review Boards. Data were collected as part of a larger dyadic daily and longitudinal study among couples. For the Time 1 survey, partners independently accessed a unique hyperlink to complete a consent form and self-report questionnaires hosted by Qualtrics Research Suite. Three attention-testing questions were distributed within this survey, and participants failing two out of three of these were excluded from the study and their data deleted. Six months and 1 year later, couples who completed the Time 1 were contacted by email to complete Time 2 and Time 3 questionnaires. Each partner received a CAN$10 gift card after completing the Time 1, Time 2, and Time 3. When both partners had completed the Time 1 survey, they were contacted by a research assistant to explain the procedure for the daily diaries. Each partner accessed a unique hyperlink received via email each evening at 6:00 p.m. with a reminder at 10:00 p.m., to complete a brief survey for 35 consecutive days. Participants were instructed to complete the survey every day before going to sleep. Compensation was prorated based on how many diaries participants completed, with a maximum of CAN$50 each in gift cards. For the longitudinal follow-ups, out of 420 participants, 388 participated in the Time 2 (92.4%) and 378 participated in the Time 3 (90.0%). For the daily diaries, the 434 participants individually completed a total of 13,134 diaries out of 15,190 (434 partners, 35 days) for a completion rate of 86.5% (*M* = 30.26 diaries out of 35).

### Measures

#### Childhood Maltreatment

CM was measured at Time 1 using the Childhood Trauma Questionnaire (CTQ; Bernstein et al., 1994; 2003). This 25-item measure retrospectively assesses the extent of emotional (five items), physical (five items), and sexual abuse (five items) as well as emotional (five items) and physical neglect (five items) over the entire “growing up” period in their own family, without reference to specific ages. Participants rated each item on a five-point scale ranging from 1 (never true) to 5 (very often true). Items are summed to produce five subscales ranging from 5 (no CM) to 25 with higher scores reflecting greater frequency of this form of CM. Items can also be summed to compute a total score ranging from 25 (no CM) to 125, a higher total score reflecting multiple chronic victimization. The CTQ demonstrates good internal consistency (α = .61–.95), good temporal stability (r = .79–.95), and good convergent validity with a structured trauma interview (Bernstein et al., 1994; 2003; Paquette et al., 2004). In the present study, Cronbach’s α for the subscales varied between .70 and .95 and the α for the total score was .94.

#### Perceived Partner Responsiveness

PPR was measured using the PPR subscale of the Relationship Intimacy Measure (Bois et al., 2013) which was designed based on the diary measure of Laurenceau et al. (1998). The four items asked both partners to rate in general in the relationship or during the course of the day the degree to which they felt understood, validated, accepted, and cared for by their partner. These items were rated on a seven-point Likert scale (1 = not at all, 7 = a lot) and summed to provide a subscale score ranging from 4 to 28, with higher scores indicating greater PPR. This subscale achieves good internal consistency (Cronbach’s α = .91 and .92; Bois et al., 2013) and good construct validity (Laurenceau et al., 1998; 2005). In the present study, Cronbach’s α were .90 at Time 1 and Time 2 and .92 at Time 3. At the daily level, the Cronbach’s α was .95 and the within person reliability of change was .91.

### Data Analyses

Descriptive and correlation analyses were computed using SPSS 27. The hypotheses were then tested using M*plus* version 8.5 (Muthén & Muthén, 1998-2017). The data and syntax can be obtained at https://osf.io/nhc34/?view_only=6abedc93266249ceb5ac602584669f14. To examine the associations between CM and PPR in the daily data, we used the aggregate measures of a person’s PPR over the 35 days to compute their mean score and a person’s root mean square of successive differences (RMSSD; Ebner-Priemer & Trull, 2011) to compute their day-to-day variability score. The RMSSD was obtained by first calculating each successive day difference between PPR scores (i.e., subtracting yesterday’s PPR score from today’s PPR score). Then, each of the values was squared and averaged across the 35 days before the square root of the total was obtained. To avoid confounding the mean scores of PPR with the day-to-day variability in PPR, both were modeled simultaneously (i.e., in a single model) in an actor-partner interdependence model (APIM) using path analyses (Kenny et al., 2006). One model was computed for the effects of the cumulative CM total score and one model for the five forms of CM included simultaneously. Relationship duration was included as a covariate.

To examine the associations between CM and PPR in the longitudinal data, dyadic latent growth curve models (LGCM) were computed within a structural equation model (Kashy et al., 2008). First, an unconditional dyadic LGCM was computed to examine fixed- and random-estimates of the intercept and the slope of PPR. The intercept represents the level of PPR at the beginning of the study and the slope represents the trajectory from Time 1 to Time 3. Second, two conditional dyadic LGCMs were performed (i.e., one model for the cumulative CM score and one model for the five forms of CM included simultaneously). These LGCM included CM as a time-invariant covariate with fixed effects to predict an individual’ own and their partner’s intercepts (initial levels) and slopes (trajectories) of PPR. As nine couples became pregnant during the longitudinal follow-up, we controlled for pregnancy in the conditional dyadic LGCM (0 = not pregnant; 1 = pregnant at Time 2 or Time 3) as well as relationship duration.

For the daily and the longitudinal models, APIM analyses were conducted because they account for the interdependence between partners and allow testing for actor effects controlling for partner effects, and for partner effects controlling for actor effects (Kenny et al., 2006). As this sample included both same- and mixed-gender/sex couples, no variable could distinguish all dyads, thus they were conceptually considered indistinguishable with each partner being randomly assigned to “partner 1” and “partner 2” and adding equality constraints on all parameters between partners (Kashy et al., 2008). All analyses were performed with the maximum likelihood parameter estimates with robust SEs and chi-square test (MLR). Attrition not due to separation and score-level missing data were handled using full information maximum likelihood (Muthén & Muthén, 1998-2017). Overall model fit was evaluated by considering together commonly used fit indices (Kline, 2015): a non-statistically significant chi-square; a comparative fit index (CFI) of .95 or higher; a root mean square error of approximation (RMSEA) below .05; and, a standardized root mean square residual (SRMR) below .10.

## Results

### Descriptive Analyses

Means, *SD*s, ranges, and correlations are presented in Table 1. Age, gender, sexual orientation, years of education, annual income, and relationship status were not significantly correlated with the mean-level over 35 days, the day-to-day variability, and Time 1, Time 2, and Time 3 PPR above *r* = .15; thus, we did not control for them in further analyses.

**Table 1.** Descriptive Statistics and Correlations Among Childhood Maltreatment and Perceived Partner Responsiveness.

	*n*	Skew. (SE)	Kurt. (SE)	*M* (*SD*)	Range %	1	2	3	4	5	6	7	8	9	10	11
1. Cumulative CM	456	2.29 (.18)	8.49 (.36)	39.06 (15.59)	25–125	**.13** ^**^	.80^***^	.88^***^	.65^***^	.76^***^	.82^***^	−.18^***^	.24^***^	−.19^***^	−.13^**^	−.15^**^
2. Physical abuse	456	4.12 (.18)	20.61 (.36)	6.62 (3.52)	5–25	.10^*^	**.14** ^**^	.68^***^	.49^***^	.54^***^	.49^***^	−.07	.18^***^	−.12^*^	−.04	−.04
3. Emotional abuse	456	1.50 (.18)	1.96 (.36)	8.87 (4.78)	5–25	.09^*^	.08	**.06**	.45^***^	.56^***^	.69^***^	−.10^*^	.21^***^	−.12^*^	−.05	−.06
4. Sexual abuse	456	4.27 (.18)	23.24 (.36)	6.27 (3.68)	5–25	.10^*^	.08	.09	**.08**	.34^***^	.32^***^	−.14^**^	.13^**^	−.16^**^	−.09	−.10
5. Physical neglect	456	2.62 (.18)	9.23 (.36)	6.95 (2.98)	5–25	.09^*^	.06	.05	.11^*^	**.05**	.63^***^	−.20^***^	.19^***^	−.21^***^	−.14^**^	−.20^***^
6. Emotional neglect	456	.95 (.18)	.47 (.36)	10.35 (4.73)	5–25	.12^*^	.05	.09	.04	.10^*^	**.16** ^***^	−.21^***^	.23^***^	−.18^***^	−.19^***^	−.20^***^
7. Mean of daily PPR	434	−1.07 (.18)	1.30 (.36)	22.85 (3.83)	7.45–28	−.09	−.05	−.06	−.06	−.13^**^	−.07	**.68** ^***^	−.49^***^	.64^***^	.68^***^	.63^***^
8. Day-to-day variability in PPR	433	.55 (.18)	−.07 (.36)	3.87 (2.03)	0–12.64	.12^*^	.12^*^	.12^**^	.05	.09	.09	−.29^***^	**.37** ^***^	−.30^***^	−.30^***^	−.20^***^
9. PPR time 1	420	−2.03 (.18)	5.01 (.36)	24.38 (3.96)	7–28	−.11^*^	−.08	−.05	−.03	−.13^**^	−.15^**^	.52^***^	−.27^***^	**.52** ^***^	.70^***^	.56^***^
10. PPR time 2	387	−1.71 (.18)	3.47 (.36)	24.48 (3.90)	8–28	−.05	−.03	−.03	.01	−.08	−.08	.45^***^	−.18^***^	.43^***^	**.41** ^***^	.65^***^
11. PPR time 3	375	−1.59 (.18)	2.38 (.36)	23.91 (4.70)	6–28	−.11^*^	−.02	−.06	−.00	−.21^***^	−.15^**^	.46^***^	−.18^**^	.41^***^	.36^***^	**.54** ^***^

*Note.* CM = childhood maltreatment; PPR = Perceived Partner Responsiveness. Correlations above the diagonal are between each of the actor variables and correlations along (in bold) and below the diagonal are between the actor and partner variables. The sample for the correlations varied between *n* = 364 and *n* = 456. The correlations take into account that participants are nested within each couple. ^*^p < .05. ^**^p < .010. ^***^p < .001.

### Daily Associations with Perceived Partner Responsiveness

We examined if a person’s cumulative CM was associated with their own and their partner’s mean level and day-to-day variability of PPR. This model provided good fit indices: χ (16) = 8.17, *p* = .944; RMSEA = .00, 90% CI = [.00, .01]; CFI = 1.00; SRMR = .04. Results are presented in Table 2 and showed that a person’s cumulative CM was related to a lower mean level of their own PPR and greater day-to-day variability in their own and their partner’s PPR. A person’s cumulative CM was unrelated to the mean level of their partner’s PPR. This model explained 5.1% of the variance in PPR’s mean level and 8.3% in PPR’s day-to-day variability.

**Table 2.** Associations Between Actor and Partner Childhood Maltreatment and Daily Perceived Partner Responsiveness (*n* = 434).

	Mean of daily PPR				Day-to-day variability in PPR			
Model 1	β	b (SE)	*p*-value	[95% *CI*]	β	b (SE)	*p*-value	[95% *CI*]
Actor cumulative CM	**−.18**	**−.05 (.02)**	**.002**	**[**−**.07,** −**.02]**	**.24**	**.03 (.01)**	**< .001**	**[.02, .05]**
Partner cumulative CM	−.08	−.02 (.01)	.077	[−.04, <.01]	**.11**	**.02 (.01)**	**.029**	**[<.01, .03]**
Relationship length	−.11	−.08 (.04)	.064	[−.17, .01]	**−.11**	**−.05 (.02)**	**.016**	**[**−**.08,** −**.01]**
Model 2	β	b (SE)	*p*-value	[95% *CI*]	β	b (SE)	*p*-value	[95% *CI*]
Actor physical abuse	.06	.06 (.07)	.332	[−.07, .19]	.03	.02 (.05)	.744	[−.08, .11]
Actor emotional abuse	.14	.11 (.07)	.094	[−.02, .24]	.04	.02 (.04)	.647	[−.06, .09]
Actor sexual abuse	−.11	−.13 (.08)	.112	[−.28, .03]	.05	.03 (.04)	.477	[−.05, .11]
Actor physical neglect	**−.14**	**−.19 (.09)**	**.040**	**[**−**.38,** −**.01]**	.05	.04 (.05)	.476	[−.06, .14]
Actor emotional neglect	**−.21**	**−.17 (.06)**	**.003**	**[**−**.28,** −**.06]**	.14	.06 (.03)	.063	[<−.00, .13]
Partner physical abuse	−.02	−.02 (.07)	.734	[−.15, .11]	.09	.05 (.04)	.193	[−.03, .14]
Partner emotional abuse	.01	.01 (.06)	.880	[−.12, .13]	.08	.03 (.03)	.282	[−.03, .09]
Partner sexual abuse	−.03	−.03 (.06)	.579	[−.15, .09]	−.01	−.01 (.04)	.851	[−.08, .07]
Partner physical neglect	−.11	−.15 (.09)	.084	[−.33, .02]	<.00	<.01 (.05)	.981	[−.09, .09]
Partner emotional neglect	.05	.04 (.05)	.477	[−.07, .14]	−.03	−.02 (.04)	.677	[−.08, .06]
Relationship length	−.11	−.08 (.04)	.067	[−.17, .01]	**−.11**	**−.05 (.02)**	**.018**	**[**−**.08,** −**.01]**

*Note.* PPR = Perceived Partner Responsiveness; CM = childhood maltreatment; β = standardized coefficients; b = unstandardized coefficients; SE = standard error; CI = confidence interval. Mean of daily PPR and day-to-day variability in PPR were modeled simultaneously. Coefficients in bolds are significant at *p* < .05.

We examined if a person’s physical abuse, emotional abuse, sexual abuse, physical neglect and emotional neglect were associated with their own and their partner’s mean level and day-to-day variability of PPR. This model provided good fit indices: χ (68) = 79.00, *p* = .170; RMSEA = .03, 90% CI = [.00, .05]; CFI = .96; SRMR = .06. Results are presented in Table 2 and showed that a person’s physical and emotional neglect were related to a lower mean level of their own PPR. All forms of CM were unrelated to the mean level of their partner’s PPR and to day-to-day variability in their own and their partner’s PPR. This model explained 9.4% of the variance in PPR’s mean level and 9.0% in PPR’s day-to-day variability.

### Longitudinal Associations with Perceived Partner Responsiveness

The unconditional dyadic LGCM provided good fit indices: *χ*^2^ (13) = 7.32, *p* = .885; CFI = 1.00; RMSEA = .00; 90% CI = [.00, .03]; SRMR = .09. PPR started at a mean level of 24.46 (SE = .23, *p* < .001) and declined significantly by −.26 (SE = .11, *p* = .017) between each time point. We examined if a person’s cumulative CM was associated with their own and their partner’s intercept and slope of PPR over time in a dyadic LGCM. This model provided good fit indices: χ (36) = 32.24, *p* = .648; RMSEA = .00, 90% CI = [.00 to .04]; CFI = 1.00; SRMR = .08. Results are presented in Table 3 and showed that a person’s cumulative CM was related to a lower initial level of their own PPR but was unrelated to the initial level of their partner’s PPR. A person’s cumulative CM was also unrelated to their own and their partner’s PPR over time. This model explained 7.7% of the variance in the initial level of PPR and 1.5% in PPR over time.

**Table 3.** Associations Between Actor and Partner Childhood Maltreatment and Perceived Partner Responsiveness Over Time (*n* = 420).

	Perceived partner responsiveness over time							
Model 1	Mean (SE)		*p*-value	[95% *CI*]	Residual variance (SE)		*p*-value	[95% *CI*]
Intercept	**27.52 (.74)**		**<.001**	**[26.08, 28.97]**	**11.00 (1.94)**		**<.001**	**[7.20, 14.80]**
Slope	−.05 (.58)		.935	[−.20, .18]	1.00 (.79)		.207	[−.02, .07]
	Intercept				Slope			
	β	b (SE)	*p*-value	[95% *CI*]	β	b (SE)	*p*-value	[95% *CI*]
Actor cumulative CM	**−.19**	**−.04 (.01)**	**.001**	**[−.07, −.02]**	<.00	<.01 (.01)	.998	[−.02, .02]
Partner cumulative CM	−.08	−.02 (.01)	.145	[−.04, .01]	−.07	−.01 (.01)	.583	[−.02, .01]
Relationship length	**−.16**	**−.11 (.04)**	**.002**	**[−.18, −.04]**	−.04	− .01 (.03)	.788	[−.07, .05]
Pregnancy	.01	.22 (.66)	.735	[−1.06, 1.51]	.09	.45 (.63)	.478	[−.79, 1.68]
Model 2	Mean (SE)		*p*-value	[95% *CI*]	Residual variance (SE)		*p*-value	[95% *CI*]
Intercept	**28.23 (.84)**		**<.001**	**[26.59, 29.88]**	**10.84 (1.90)**		**<.001**	**[7.12, 14.55]**
Slope	.08 (.63)		.901	[−1.15, 1.31]	1.12 (.76)		.144	[−.38, 2.61]
	Intercept				Slope			
	β	b (SE)	*p*-value	[95% *CI*]	β	b (SE)	*p*-value	[95% *CI*]
Actor physical abuse	.03	.03 (.07)	.654	[−.10, .17]	.02	.01 (.05)	.890	[−.10, .11]
Actor emotional abuse	.09	.07 (.06)	.300	[−.06, .19]	.20	.05 (.03)	.170	[−.02, .11]
Actor sexual abuse	**−.13**	**−.13 (.06)**	**.035**	**[−.24, −.01]**	.02	.01 (.05)	.898	[−.09, .10]
Actor physical neglect	**−.15**	**−.19 (.09)**	**.037**	**[−.37, −.01]**	.05	.02 (.06)	.726	[−.09, .13]
Actor emotional neglect	−.11	−.09 (.06)	.133	[−.20, .03]	**−.29**	**−.07 (.03)**	**.029**	**[−.13, −.01]**
Partner physical abuse	−.10	−.11 (.07)	.156	[−.25, .04]	.18	.06 (.06)	.312	[−.05, .17]
Partner emotional abuse	.15	.11 (.08)	.154	[−.04, .27]	−.15	−.04 (.05)	.425	[−.12, .05]
Partner sexual abuse	.04	.04 (.06)	.487	[−.07, .15]	−.04	−.01 (.03)	.694	[−.07, .05]
Partner physical neglect	−.03	−.03 (.10)	.759	[−.23, .17]	−.23	−.09 (.05)	.076	[−.19, .01]
Partner emotional neglect	**−.17**	**−.13 (.06)**	**.036**	**[−.25, −.01]**	.17	.04 (.04)	.243	[−.03, .11]
Relationship length	**−.16**	**−.11 (.04)**	**.002**	**[−.18, −.04]**	−.04	−.01 (.03)	.760	[−.07, .05]
Pregnancy	.01	.25 (.62)	.691	[−.97, 1.47]	.08	.47 (.63)	.462	[−,77, 1.70]

*Note.* CM = childhood maltreatment; β = standardized coefficients; b = unstandardized coefficients; SE = standard error; CI = confidence interval; Coefficients in bolds are significant at *p* < .05.

We examined if a person’s physical abuse, emotional abuse, sexual abuse, physical neglect and emotional neglect were associated with their own and their partner’s intercept and slope of PPR in a conditional dyadic LGCM. This model provided good fit indices: χ (112) = 109.05, *p* = .561; RMSEA = .00, 90% CI = [.00, .03]; CFI = 1.00; SRMR = .07. Results are presented in Table 3 and showed that a person’s sexual abuse and physical neglect were related to lower initial level of their own PPR. A person’s emotional neglect was related to lower initial level of their partner’s PPR and to a decrease over time in their own PPR. This model explained 11.8% of the variance in the initial level of PPR and 10.2% of the variance in PPR over time.

## Discussion

PPR plays a critical role in a person’s mental and physical health as well as romantic relationship functioning (Laurenceau et al., 2005; Tasfiliz et al., 2018). This study examined whether CM may represent a developmental factor that hampers the perception of partner responsiveness using mean-level of PPR over 35 days, day-to-day variability, and initial levels and trajectories of PPR over 1 year. The findings indicated that a person’s cumulative CM was not only related to their own lower PPR at a given time point, but also to greater day-to-day variability in their own and their partner’s PPR. In addition, one specific form of CM, emotional neglect, was related to a sharper decrease over time in participants’ own PPR.

### Cross-Sectional Associations with Perceived Partner Responsiveness

The first main finding was that when a person reported cumulative CM, they felt less understood, validated, and cared for by their partner over the 35 days and at the beginning of the 1-year follow-up. These results replicate the cross-sectional findings of Vaillancourt-Morel et al. (2019) which showed that an individual’s cumulative CM was related to their own lower levels of PPR. It also supports the numerous complex effects CM may have on intimate relationships leading to unsatisfying, unstable, or conflictual relationships (Whisman, 2006; Zamir, 2021). Trauma theoretical models (Briere & Scott, 2014) suggest that the perception of partner understanding, validation, and care may be particularly biased or distorted in individuals having experienced multiple forms of CM in which a caretaker, who was supposed to protect them, abused, neglected, and invalidated them. Thus, even a caring and loving interaction may be fueled by past CM feelings of betrayal, powerlessness, and perplexity (Briere, 2002), leading to potential misinterpretations of the partner’s behaviors. Alternatively, this perception of partner responsiveness may also represent reality, as partners of individuals reporting CM may show insufficient empathic responses. Past studies have shown that CM is related to an increased risk of repetition of abusive relationships (DiLillo et al., 2009; Godbout et al., 2019).

Our findings also expand Vaillancourt-Morel et al.’s (2019) results as the models including all forms of CM simultaneously showed that a person’s physical and emotional neglect were uniquely related to a lower level of their own PPR over 35 days and at baseline of the 1-year follow-up, and that a person’s sexual abuse was uniquely related to a lower level of their own baseline PPR in the follow-up. Even if few studies have tried to tease out the unique effects of each form of CM, these results are in line with past research indicating that emotional and sexual maltreatment are more deleterious for psychological and interpersonal outcomes than other forms of CM (Gibb et al., 2001; Senn & Carey, 2010; Vaillancourt-Morel et al., 2021). Neglect represents the failure of the caretaker to care, emotionally and physically, for the child, and intrafamilial sexual abuse often occurs coupled with affection, attention, and rewards. The nature of these maltreatments and the mixed emotions felt toward the abusive or neglecting caretaker may explain why, in later intimate relationships, the ambivalent affect and cognitions of these specific CMs are particularly triggered when the partner shows validation and care.

### Associations with Variability in Perceived Partner Responsiveness

The second main finding was that a person’s cumulative CM was related to greater day-to-day variability in their own and their partner’s PPR. The instability in victims’ perception of PPR is a novel contribution, but is in line with past studies reporting an association between CM and dichotomous thinking that alternates between idealization and devaluations of others (Showers et al., 2006; Twomey et al., 2000). Our results showed that it is not a specific form of CM that is uniquely related to this greater day-to-day variability, but that it is the accumulation of multiple forms of CM which is in line with the complexity of symptoms related to the notion of complex trauma (Briere, 2002; Courtois, 2004). For a child, integrating the attachment to a caretaker, which is the source of protection and love, with this caretaker’s abusive and neglecting behaviors requires extraordinary defense mechanisms (e.g., splitting, dissociation). These ways of dealing with the traumatic experience may be reactivated in romantic relationships and explain this greater day-to-day instability in their own perception of their partner responses (MacIntosh, 2017). Combining the view of a partner as loving with the normal day-to-day frustrations and disappointments may be particularly challenging for victims of complex trauma, leading to the reactivation of CM-related perceptions that alternate between, 1 day, a responsive partner, and, another day, an insensitive and rejecting partner. Another plausible hypothesis is that variability in PPR represents reality, as individuals reporting cumulative CM may choose partners that are more unstable in their empathic responses, as CM is related to more unstable and violent relationships (Colman & Widom, 2004; Godbout et al., 2019).

The association between a person’s cumulative CM and their partner’s greater instability in their PPR is in line with secondary trauma effects of CM on romantic partners (Nelson & Wampler, 2000) and past studies reporting lower relationship satisfaction and lower accuracy to read negative emotions in partners of individuals reporting CM (Maneta et al., 2014; Whisman, 2014). CM may be associated with partners’ perception of PPR via feelings that parallel the victim’s mixed emotional responses and cognitive biases (MacIntosh, 2017). This effect on partners may also reflect a more unstable environment, where both partners show high variability in their perception of the other. On the other hand, as CM is related to deficits in the ability to empathize with others (Locher et al., 2014) and greater day-to-day variability in emotions (Infurna et al., 2015), individuals reporting CM may show more emotional lability and greater lapses in emotional availability toward the partner. Thus, partners may adequately perceive greater variability in the empathic responses of individuals reporting CM.

### Associations with Trajectories of Perceived Partner Responsiveness over 1 Year

Even if cumulative CM was unrelated to PPR over time, therefore not supporting our hypothesis, a person’s emotional neglect was associated with a sharper decrease over time in their own PPR. Although this was the only form of CM significantly related to PPR over time, this results is in line with past studies that have shown that emotional neglect has a detrimental effect on relationship and sexual satisfaction over the course of a romantic relationship (DiLillo et al., 2009; Vaillancourt-Morel et al., 2021). Even if emotional neglect is far more subtle than abuse, it is associated with worse outcomes (Hildyard & Wolfe, 2002). When a child’s emotional needs are consistently dismissed, the child is left with a biased view of self and others—a sense of self as empty and needy and of others as a source of rejection (Bowlby, 1969; Briere, 2002). As the relationship progresses and given that PPR naturally declines in romantic relationships, individuals with a history of emotional neglect may experience more difficulties dealing with lower empathic responses from the partner, which may re-enact the view of a significant other as rejecting and unavailable for the undeserving self (MacIntosh, 2017).

### Limitations

This study has limitations that should be considered when interpreting the results. First, although the study used a dyadic daily diary and longitudinal methodology, the correlational design and the lack of statistical control for other potential confounding factors including the global family and social context make it impossible to determine causal relations. Moreover, we did not include potential mediators (e.g., attachment, emotion regulation, and depressive symptoms) that could explain the CM-PPR association and this is an important avenue for future research. Second, the generalizability of our results is potentially limited due to convenience sampling. Our findings must be understood in the context of the sample characteristics, that is, relatively young, sexually active with couples little cultural diversity. Moreover, this study included a wide range of relationship lengths and although we controlled for this variability, we did not examine whether our associations were different across the various stages of a relationship. Third, even if daily diaries have many strengths, all perceptions of partner responsiveness were collected via self-report measures, which only represent individuals’ self-perceptions. Thus, it was not possible to determine whether the associations between CM and PPR were fueled by biased perceptions induced by past CM or a reflection of actual partner behaviors. As such, we cannot comment on the partner’s behaviors per se.

### Implications for Research and Practice

Findings underscore the importance of considering the complexity of the effects that CM may have on romantic relationships taking into account day-to-day variability as well as evolution over time in the perception of the partner, that is, some repercussions that were not apparent before may emerge over time or change from 1 day to another. Thus, CM represents an important contributing or maintenance factor for distressed couples. Researchers and clinicians might attempt, in a first step, to disentangle what is coming from the inner world of the individuals reporting CM and what is coming from the partner’s difficulties in responding empathically (Briere, 2002; MacIntosh, 2017). Future studies combining self-report measures and coders’ observations using in-lab observational methods would offer a more comprehensive understanding of the effects of CM on perceptions. In clinical practice, it may be valuable to explore with the clients their perceptions of partner responsiveness and the source of it—others’ real behavior or past relationships that were indeed non-empathic and highly variable (MacIntosh, 2013, 2019). Given the intimate and vulnerable context of a therapeutic setting, the CM-related perception may also emerge toward the therapist and represent meaningful moments toward recovery. Trauma-informed couple therapy may help couples integrate the trauma into a meaningful perspective, challenge the victim’s and their partner’s perception of others, and ultimately, help them understand that their perceptions are more relevant to their CM than to the interpersonal context in which it is triggered.
